# Distant genetic variants of *Anaplasma phagocytophilum* from *Ixodes ricinus* attached to people

**DOI:** 10.1186/s13071-023-05654-y

**Published:** 2023-02-28

**Authors:** Paulina Maria Lesiczka, Kristyna Hrazdilova, Václav Hönig, David Modrý, Ludek Zurek

**Affiliations:** 1 CEITEC–University of Veterinary Sciences, Brno, Czech Republic; 2 Department of Veterinary Sciences, Faculty of Agrobiology, Food and Natural Resources, Czech University of Life Sciences, Prague, Czech Republic; 3 Department of Chemistry and Biochemistry, Mendel University, Brno, Czech Republic; 4 Faculty of Medicine in Pilsen, Biomedical Center, Charles University, Pilsen, Czech Republic; 5 Biology Centre, Institute of Parasitology, Czech Academy of Sciences, České Budějovice, Czech Republic; 6 Department of Botany and Zoology, Faculty of Science, Masaryk University, Brno, Czech Republic; 7 Department of Microbiology, Nutrition and Dietetics, Faculty of Agrobiology, Food and Natural Resources, Czech University of Life Sciences, Prague, Czech Republic

**Keywords:** *Anaplasma phagocytophilum*, *Ixodes ricinus*, Anaplasmosis, Genetic diversity, Infectious diseases

## Abstract

**Background:**

Although the tick-borne pathogen *Anaplasma phagocytophilum* is currently described as a single species, studies using genetic markers can distinguish groups of variants associated with different hosts, pathogenicity, zoonotic potential and biotic and geographic niches. The objective of our study was to investigate the genetic diversity of *A. phagocytophilum* and *Ixodes ricinus* ticks attached to people.

**Methods:**

In collaboration with a commercial diagnostic company, a total of 52 DNA samples were obtained from ticks that tested positive for *A. phagocytophilum* by quantitative PCR. The genetic profile of each sample was determined using the *groEL* and *ankA* genes. Identification of the tick species was confirmed by partial sequencing of the *COI* subunit and a portion of the *TROSPA* gene.

**Results:**

All 52 ticks were identified as *I. ricinus*. Two protocols of nested PCR amplifying 1293- and 407-bp fragments of *groEL* of *A. phagocytophilum* yielded amplicons of the expected size for all 52 samples. Among all sequences, we identified 10 unique genetic variants of *groEL* belonging to ecotype I and ecotype II. The analysis targeting *ankA* was successful in 46 of 52 ticks. Among all sequences, we identified 21 unique genetic variants phylogenetically belonging to three clusters.

**Conclusions:**

Our results indicate that ticks attached to people harbor distant genetic variants of *A. phagocytophilum*, some of which are not recognized as zoonotic. Further studies are needed to determine the risk of human infection by genetic variants other than those designated as zoonotic.

**Graphical Abstract:**

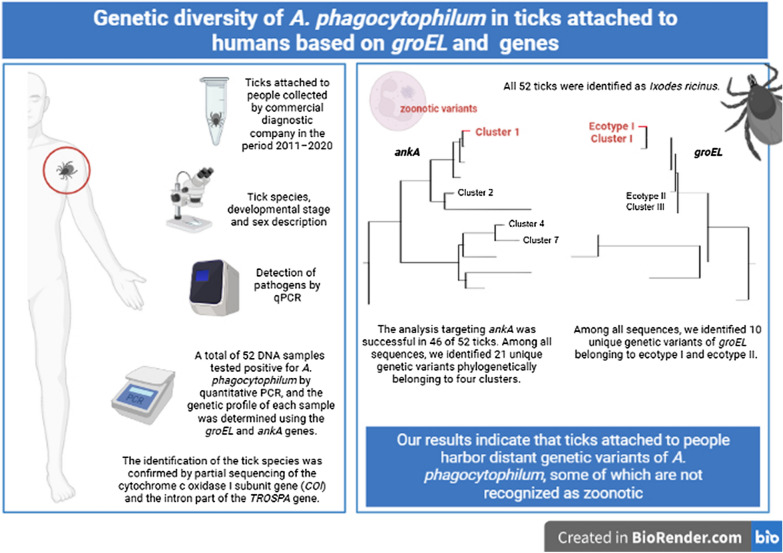

**Supplementary Information:**

The online version contains supplementary material available at 10.1186/s13071-023-05654-y.

## Background

*Anaplasma phagocytophilum* is a Gram-negative bacterium responsible for human granulocytic anaplasmosis (HGA) in humans, tick-borne fever (TBF) in ruminants, equine granulocytic anaplasmosis (EGA) in horses and granulocytic anaplasmosis in dogs and cats [1]. In the Palearctic region, the dominant genetic variants of *A. phagocytophilum* are transmitted by *Ixodes ricinus* and *Ixodes* *persulcatus*, while in the Nearctic region *Ixodes scapularis* and *Ixodes* *pacificus* are the known vectors of this bacterium [2, 3].

Genetic variants of *A. phagocytophilum* in North America and Europe differ in host preference and clinical symptoms, with TBF and EGA dominant in Europe and HGA dominant in North America [4, 5]. The genetic diversity of European strains of *A. phagocytophilum* of different origins has been demonstrated by phylogenetic analyses of genes such as *groEL* [6–8], *ankA* [4] and *msp4* [9]. Different target genes led to different names of the genetic variants and consequently different terminology, such as ecotype (*groEL*), cluster (*ankA* and *groEL*) and genotype (*msp4*) [4, 10].

The heat shock operon groESL contains two genes encoding the chaperone proteins groES and groEL, respectively, as well as the intergenic region. A 5′ fragment of *groEL* has been widely used for genotyping *A. phagocytophilum*, especially those from Europe [11–13]. Currently, based on *groEL* genotyping studies, four ecotypes and eight clusters with different pathogenicity and geographical origin are distinguished [7, 8].

The variable *ankA* gene encodes the ankyrin repeat-containing protein AnkA (153–160 kDa). The *ankA* differentiates variants corresponding with the species of their animal hosts and exhibits higher sequence variability compared to that of 16S ribosomal DNA, *groEL* and *msp4* [3]. Studies have found an association among genetic variants and vertebrate hosts, tick vectors and geographic locations such that regardless of the gene used for analysis, infected humans, whether in Europe or the Americas, appear to share related strains belonging to the same genetic group [5, 14].

The number of HGA cases reported in the USA has been steadily increasing since reporting of the disease was initiated, increasing from 348 in 2000 to 5762 cases in 2017 (https://www.cdc.gov/anaplasmosis/stats/index.html). In Europe, the total annual number of HGA cases has not exceeded 300 [2, 15]. Several HGA cases have also been reported in Canada, Russia, China, Taiwan, South Korea and Japan (for review, see [3]). According to the National Institute of Public Health in the Czech Republic, 53 cases of HGA were reported between 2007 and 2017; however, in the seroprevalence study from 2014, specific antibodies were detected in 34 of 314 individuals tested [16]. More recently, 12.6% of 103 patients with clinical symptoms persisting after antibiotic treatment of diagnosed Lyme disease were positive for *A. phagocytophilum* immunoglobulin G antibodies [17]. Similar rates of seroprevalence were reported in other European countries, including Norway, Sweden and Poland, where *Anaplasma* antibodies were detected in 11.0%, 12.0% and 11.8% of the general population, respectively [17]. The disease is likely to be greatly underdiagnosed due to its nonspecific flu-like symptoms, such as fever, headache and myalgias, which usually resolve without treatment [1]. If the immune system fails and the infection is left untreated, the disease can cause life-threatening symptoms, such as respiratory failure, severe gastrointestinal bleeding, renal failure and liver damage [18]. Although the number of surveillance reports of *A. phagocytophilum* in tick vectors is increasing worldwide, the epidemiological risk of infection by this pathogen is underestimated [19]. Detection of pathogens in blood-feeding ticks shows the risk of human exposure better than studies on foraging ticks [20]. In this context, the main objective of our study was to investigate the genetic diversity of *A. phagocytophilum* in ticks attached to people in the Czech Republic nationwide.

## Methods

### Tick identification

**Fig. 1:**
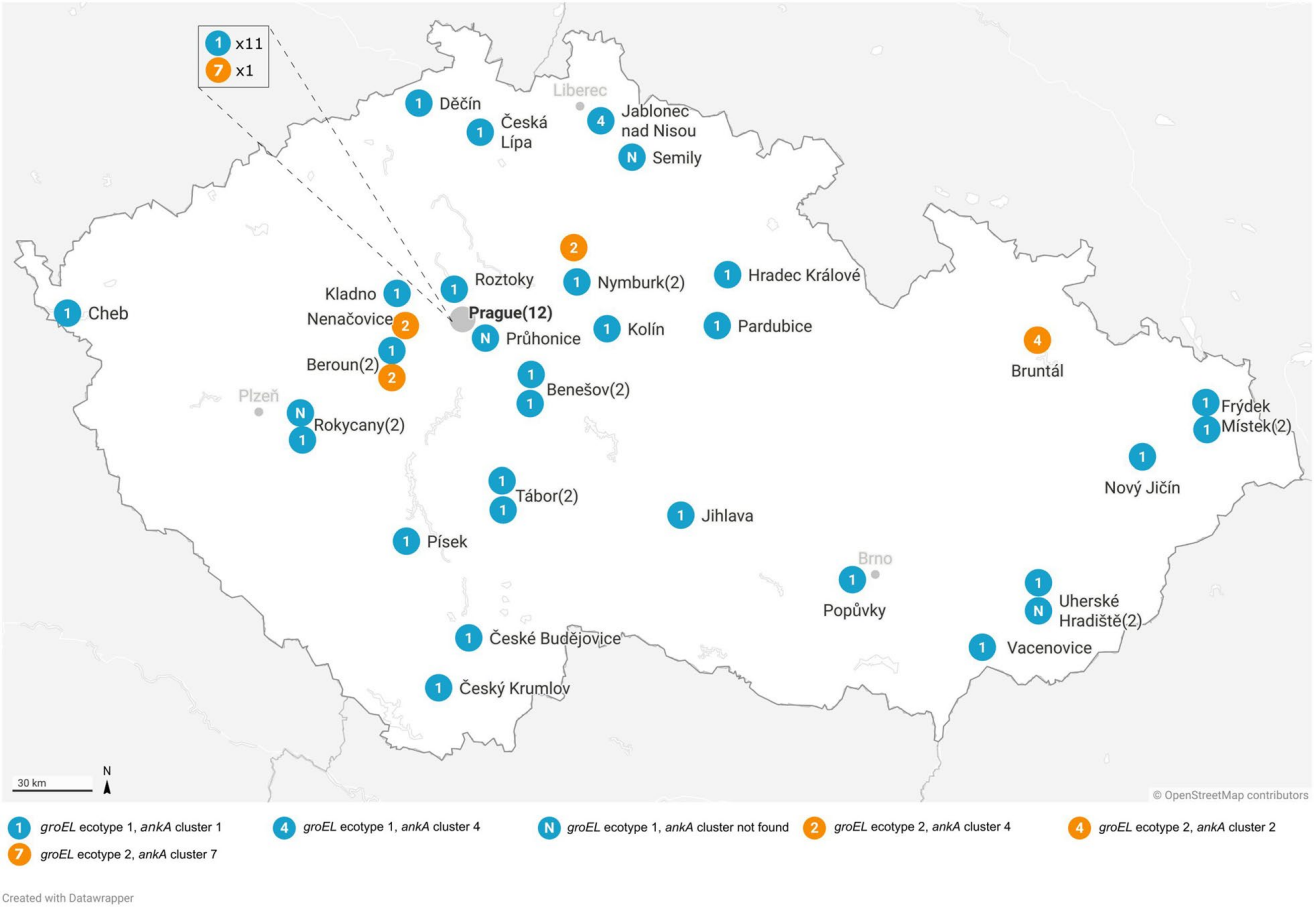
Map of the collection sites of the 46 samples of ticks that tested positive for *Anaplasma phagocytophilum* across the Czech Republic. The collection site for 6 ticks was not known. Numbers in parentheses represent the number of ticks from the same locality

A total of 52 DNA samples of ticks that tested positive for *A. phagocytophilum* by quantitative PCR [21] and were attached to people at collection were acquired in collaboration with a commercial diagnostic company that offered pathogen detection in ticks sent by the general public (Fig. 1). Tick species, developmental stage and sex were determined by the specialist from the collaborating institution. Samples were collected in the period 2011–2020, and each sample was individually homogenized, following which nucleic acids were isolated by the ExiPrep Plus Viral DNA/RNA Kit using the Exiprep 16 Plus nucleic acid extraction system (Bioneer, Daejeon, Republic of Korea).

The identification of the tick species was confirmed by partial sequencing of the cytochrome *c* oxidase I subunit gene (*COI*) and the intron part of the* TROSPA* gene, as described by [22]. The amplification of the *COI* was performed in a total reaction volume of 20.0 µl comprising the Phusion Green Hot Start II High-Fidelity PCR Master Mix (Thermo Fisher Scientific, Waltham, MA, USA) following the manufacturer’s instructions at an annealing temperature of 55 °C. Amplification of* TROSPA* was performed in a total reaction volume of 25.0 µl comprising 2× PCR BIO Taq Mix Red (PCR Biosystems Ltd., London, UK), following the manufacturer instructions at an annealing temperature of 65 °C. Amplicons were visualized, processed and sequenced as described in the following section. Sequence identity was determined by BLASTn analyses of the GenBank database at NCBI.

### Amplification of *groEL* and *ankA*

To determine the *groEL* ecotype, nested PCR was performed targeting a 407-bp portion of the variable fragment of *groEL*, as described previously [7, 23]. Whenever possible, a longer fragment of *groEL* (1297 bp) was amplified following the nested PCR protocol [6] using slightly modified primers [24]. Subsequently, the samples were analyzed using two nested PCR assays aimed at the *ankA* gene (Additional file 1: Table S1). The first assay (herein called protocol 1) amplified an approximately 530-bp fragment of the *ankA* gene, and the second assay (protocol 2) amplified an approximately 500 bp. All PCRs were performed using the commercial master mix (2× PCR BIO Taq Mix Red; PCR Biosystems Ltd.) following the manufacturer’s instructions. A total volume of 25.0 μl was prepared for each reaction, comprising 12.5 μl of the master mix, 10.0 pmol of each primer, 2.0 μl of template DNA or 1.0 μl of PCR product from the first round in the case of nested PCR, with PCR water used for the remaining volume. All PCR products were visualized on an 1.5% agarose gel with the Midori Green Advance DNA System (Nippon Genetics Europe GmbH, Düren, Germany). Products of the expected size were purified using the Gel/PCR DNA Fragments Extraction Kit (Geneaid Biotech Ltd., New Taipei City, Taiwan) and sequenced at the Macrogen capillary sequencing services (Macrogen Europe, Amsterdam, the Netherlands) using the amplification primers.

### Sequence and phylogenetic analysis

All sequences were assembled, edited and analyzed using the Geneious Prime® software version 2022.0.1 and compared to those available in the GenBank database by BLASTn analysis (http://blast.ncbi.nlm.nih.gov/Blast.cgi). For individual ticks, wherever possible, sequences from two separate assays that targeted overlapping regions of the *ankA* gene were assembled into a single sequence (approx. 800 bp). One sample that yielded mixed chromatogram signals was cloned using the pGEM®-T Easy Vector System (Promega, Madison, WI, USA). Cloned plasmid DNA was purified from the bacterial culture using the GenElute™ Plasmid Miniprep Kit (Sigma-Aldrich, St. Louis, MO, USA) and sequenced using the universal SP6 primer.

The phylogeny was computed separately for *groEL* and *ankA*. For *groEL*, phylogenetic analysis was performed on 87 sequences from the GenBank representing all four described ecotypes [8] and eight clusters [7] together with 10 unique sequences from this study and two sequences of *Anaplasma platys* used as an outgroup. The *ankA* gene phylogenetic analysis was performed on 74 sequences from the GenBank representing different clusters together with 21 unique sequences from this study and sequences of *Anaplasma marginale* as an outgroup.

Due to an uneven length of sequences, the alignments were calculated in two steps by the MAFFT algorithm, using “Auto” strategy for sequences > 1000 nt and function –add for implementing sequences < 1000 nt to the alignment. The phylogenetic trees were inferred by the maximum likelihood method by IQTREEv. 1.6.5 [25]. The best-fit evolution model was selected based on the Bayesian information criterion (BIC) computed by implemented ModelFinder [26]. Branch supports were assessed by the ultrafast bootstrap (UFBoot) approximation [27] and by the SH-like approximate likelihood ratio test (SH-aLRT) [28]. Trees were visualized and edited in FigTree v1.4.1 and Inkscape 0.91. The map of localities was constructed in the Datawrapper online software (https://app.datawrapper.de/).

## Results

Ticks were identified by morphology and sequencing of* TROSPA* and* COI* (data not shown). All 52 ticks were identified as *I. ricinus* and no other* Ixodes* species, including *I. inopinatus*, were found. Two protocols of nested PCR, amplifying 1293- and 407-bp fragments of *groEL*, respectively, yielded amplicons of the expected size for all 52 samples: 19 (6.3%) adults (14 females, 5 males, respectively) and 31 (10.3%) nymphs (Table 1); for two ticks, data on sex and stage development were not available. In total, eight short and 44 long sequences of *groEL* were obtained. Inspection of the chromatograms did not reveal any multiple peaks suggestive of mixed infections. Among all sequences, we identified 10 unique genetic variants with 97.9–99.9% sequence identity. The main variant (GT15) was detected in 39 individuals (75%), followed by four other variants, each detected in two ticks. The remaining five genetic variants were represented by a single individual (Fig. 1; Table 2). BLAST analysis was performed for each genetic variant. Representative sequences were deposited to the NCBI GenBank database under the accession numbers OP265397-OP265406.

**Table 1:** Genetic variants of *Anaplasma phagocytophilum* detected in ticks attached to people in the Czech Republic

Development stage of tick	Sex	*N*	*groEL*		*ankA*	
			No. of sequences	Ecotype	No. of sequences	clusters
Adult	Female	14	14	I, II	14	1, 4
	Male	5	5	I, II	5	1, 4
Nymph		31	31	I, II	23	1, 2, 4, 7
Unknown		2	2	I, II	2	1, 4

**Table 2:** Genetic variants of *A. phagocytophilum* detected in ticks attached to people in the Czech Republic

*groEL* ecotype	*groEL *variant	No. of sequences	*ankA* variant	No. of sequences	*ankA* cluster
I	GT15	39	GT152	6	1
			GT75	4	
			GT47	6	
			GT32	4	
			GT54	2	
			GT77	2	
			GT148	1	
			GT152	1	
			GT240	1	
			GT267	1	
			GT276	1	
	GT139	2	GT139	2	
	GT300	1	GT300	5^a^	
	GT294	1	GT294	3^a^	
	GT213	1	GT213	1 (2)^b^	7
	GT135	1	GT135	1	4
II	GT250	2	GT250	1	
	GT58	2	GT58	1	2
			GT270	1	
	GT184	2	GT184	1	
	GT115	1	GT115	1	

^a^Samples identical with the GT15 *groEL* genetic variant

^b^Sample with mixed chromatogram, two sequence variants obtained after cloning

The analysis targeting the *ankA* gene was successful in 46 of 52 ticks, with PCR yielding products of the expected size in 38 (455–530 bp) and 33 (470–574 bp) ticks using protocol 1 and 2, respectively (Fig. 1; Table 2). In 24 ticks, products from both assays were obtained, which enabled the assembly of longer sequence. Inspection of chromatograms of the *ankA* gene revealed multiple peaks, indicating mixed infection in one tick (GT213). Sequencing of five clones from this individual tick yielded two *A. phagocytophilum* variants with 98.7% identity (differences observed in 7 nt: 3 silent and 4 missense mutations). Among all sequences, we identified 21 unique genetic variants with 62.0–99.9% sequence identity. Representative sequences from the study were deposited to the NCBI GenBank database under the accession numbers OP265407-OP265426.

### Phylogenetic analysis

**Fig. 2:**
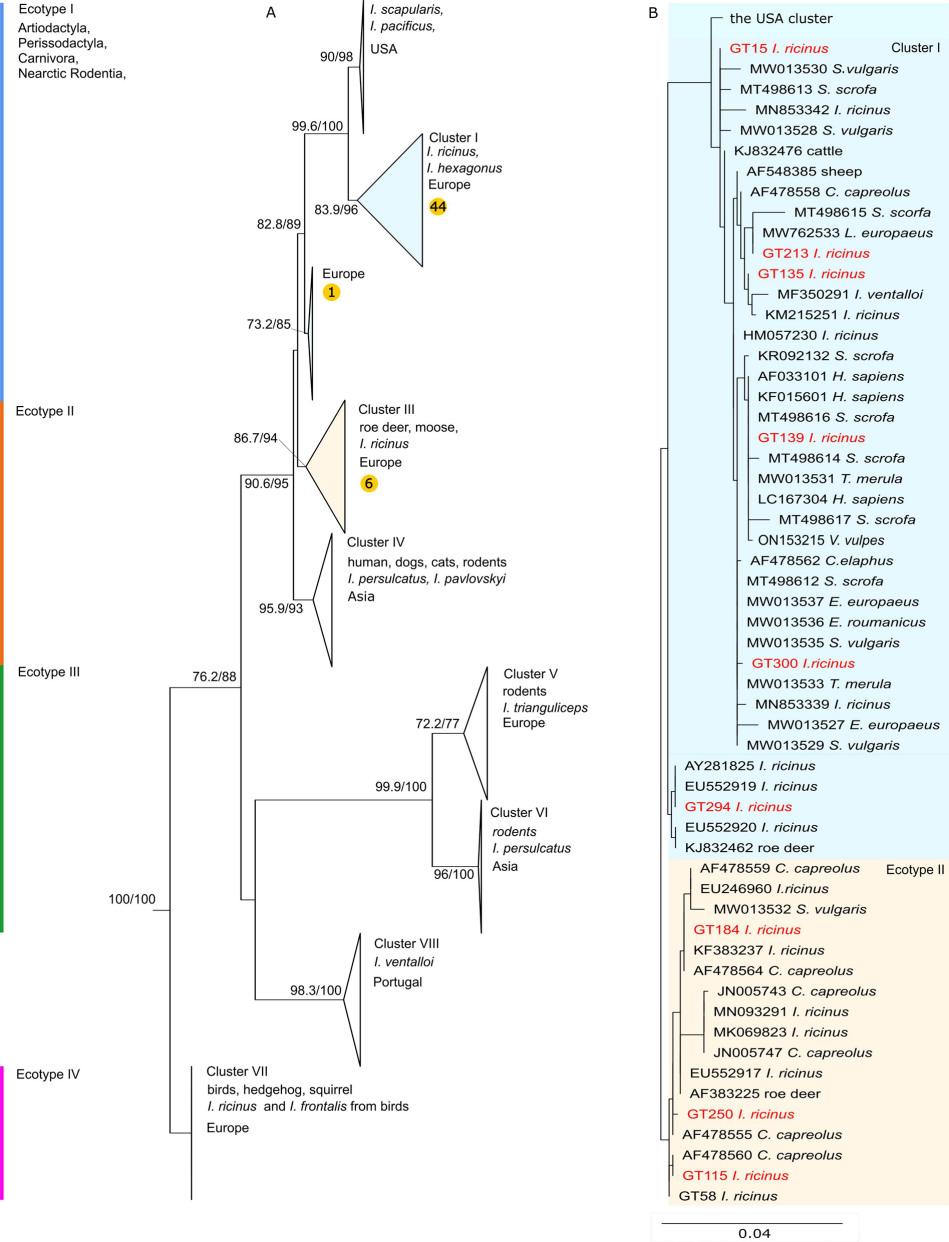
Maximum likelihood phylogenetic tree based on the groEL gene (* groEL*) of *A. phagocytophilum* (**a**). The bootstrap values (SH-aLRT/UFB) above the 80/95 threshold are displayed. Sequences of *Anaplasma platys* used as an outgroup are not displayed. The designation of the* groEL* clusters and ecotypes is given based on Jaarsma et al. [7] and Jahfari et al. [8], respectively. The highlighted clusters representing the ecotype I and ecotype II are displayed in detail in (**b**) where sequences acquired from the GenBank database are marked by their accession number and the host. Sequences obtained from this study are shown in red. SH-aLRT/UFB, SH-like approximate likelihood ratio test

In the present study, we followed the classification of *A. phagocytophilum* based on the partial *groEL* gene sequences, introduced by Jahfari et al. [8] and extended by Jaarsma et al. [7]. In the phylogenetic analysis, three well-supported clades were distinguished. The largest clade consists of two ecotypes, I and II (Fig. 2). The ecotype I is monophyletic and contains a wide range of isolates from the USA and Europe collected from humans, horses, dogs and domestic ruminants as well as wild boar and urban wildlife. Ecotype II consists of two monophyletic clusters with a high level of bootstrap support. However, ecotype II is not monophyletic, and clusters III and IV clearly differ in geographical distribution, host and vector species preference. Sequences obtained from specimens from cluster III originating from Europe were found in *I. ricinus* and deer. Cluster IV contains specimens from Asia, including isolates from a human, *I. persulcatus* and rodents (Additional file 2: Fig. S1). All sequences from our study fell into this largest clade (Fig. 2). Forty-five isolates were in two clusters belonging to ecotype I, and the remaining six isolates belong to cluster III within ecotype II (Fig. 2; Additional file 2: Fig. S1).

The second well distinguishable clade consists of isolates of ecotype III with clusters V and VI. Cluster V is represented by sequences from rodents and *Ixodes trianguliceps* from Europe and Western Siberia, and cluster VI is composed of sequences from rodents and *I. persulcatus* from Asia. These two clusters are closely related to cluster VIII [7], representing isolates from *Ixodes ventalloi* exclusively. The last clade is represented by a small number of sequences in ecotype IV/ cluster VII. None of the genetic variants detected in our study belonged to ecotype III and IV.

**Fig. 3:**
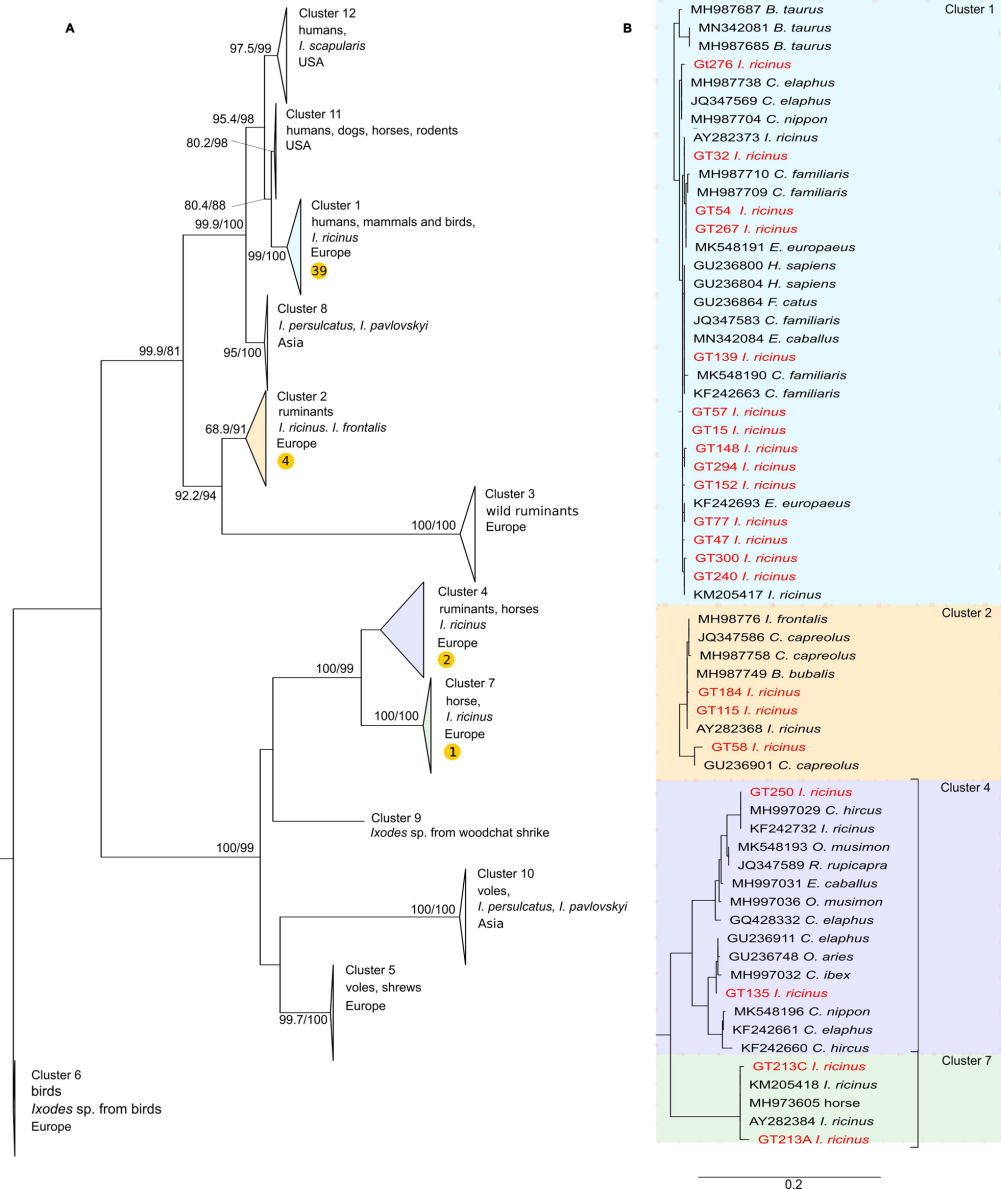
Maximum likelihood phylogenetic tree based on the ankA gene (*ankA*) of *A. phagocytophilum* (**a**). The bootstrap values (SH-aLRT/UFB) above the 80/95 threshold are displayed. The sequence of* A. marginale* used as an outgroup is not displayed. The designation of* ankA* clusters is given according to Langenwalder et al. [29] and Rar et al. [3]. The highlighted clades representing clusters 1, 2, 4 and 7 are shown in detail in **b**). Sequences acquired from the GenBank database are marked by their accession number and the host. Sequences from this study are shown in red

To describe the results of phylogenetic analysis based on the *ankA* gene, we followed the classification used by Rar et al. [3]. In our analysis, three well-supported clades were formed. The first clade consists of six clusters originating from Europe, Asia and the USA (Fig. 3; Additional file 3: Fig. S2). The specimens originating from the USA are present in clusters 11 and 12. These clusters are sister groups to *ankA* cluster 1. European cluster 1 is the most diverse and contains all of the sequences from humans, small, medium and large mammals and *I. ricinus*. Thirty-nine of our sequences were placed in this cluster 1 (Table 2). The Asian group is referred to as cluster 8 and is represented by the sequences obtained from hybrids of *I. persulcatus* × *Ixodes pavlovskyi*. The *ankA* clusters 2 and 3, also containing *A. phagocytophilum* of the European origin, are mainly represented by specimens from wild and domestic ruminants and *I. ricinus*. Four of our sequences were classified in cluster 2.

The second, well supported clade consists of five clusters. Cluster 9 is represented by a single sequence from a tick collected from a woodchat shrike (*Lanius senator*) and it is related to clusters 4 and 7 with sequences from wild and domestic ruminants and *I. ricinus* from Europe (Fig. 3; Additional file 3: Fig. S2). Two of our sequences belong to cluster 4, and two sequences from a single cloned sample fall into cluster 7. The other branch of the second clade is formed by clusters 5 and 10 from Asia and Europe. Sequences belonging to these clusters have been detected in *I. persulcatus*,* I. pavlovskyi*, their natural hybrids and rodents (Fig. 3).

The third and smallest clade contains only a single cluster 6 with sequences from birds and *Ixodes* spp. from birds (Fig. 3; Additional file 3: Fig. S2).

## Discussion

To our knowledge, this is the first elaborated study demonstrating the genetic diversity of *A. phagocytophilum* in ticks attached to humans based on *groEL* and *ankA* together. Little data are available on *A. phagocytophilum* detected in ticks feeding on humans, with current information coming from studies in Poland [30], Italy [31, 32], Romania [33] and Scotland [34]. In Europe, *A. phagocytophilum* is transmitted mainly by the tick *I. ricinus*. This tick has three feeding developmental stages, each with different host preferences, which in turn affects the range of pathogens ingested during each blood meal. Consequently, later developmental stages are more likely to carry the pathogens. The three-host life strategy of *I. ricinus* affects the transmission of *A. phagocytophilum,* which is shown in our study as well as in a study performed on questing ticks [31].

Despite the increasing number of studies on the genetic diversity of *A. phagocytophilum*, there is still insufficient data for a clear understanding of the geographic distribution, host preferences and pathogenicity to humans of the different genetic variants [24]. During the last decade, multilocus approaches gained attention as a promising tool that can help gain a better understanding of the epidemiology of *A. phagocytophilum* infections. In our study, we chose two genes and performed both analyses on the same samples. The association between *ankA* and *groEL* clusters was recently suggested by Rar et al. [3], who described concordance between specific clusters/ecotypes. In our study, 10 *groEL* and 21 *ankA* unique variants were detected, and subsequent phylogenies showed only a partial correlation between *groEL* ecotypes and *ankA* clusters. The *groEL* ecotype I has the broadest host range, and the vector *I. ricinus* has a wide distribution in the western Palearctic region and includes all genetically characterized strains detected from humans in Europe. Based on our results, *groEL* ecotype I genetic variants are represented in *ankA* clusters 1, 4 and 7, whereas *groEL* ecotype II corresponds to *ankA* clusters 2 and 4; these results support the hypothesis put forward by Rar et al. [3]. However, it should be noted that *ankA* cluster 4 consists of variants of *A. phagocytophilum* belonging to *groEL* ecotypes I and II. The *ankA* gene may be a virulence factor, and it has been suggested that it is involved in host adaptation underlying diversifying selection [2]. This may have direct implications for the observed genetic variability of this gene in *A. phagocytophilum*. The other explanation for the observed discrepancy is possible co-infection of different genetic variants of *A. phagocytophilum* in a tick sample. However, with the exception of one tick, double peaks indicative of co-infection were not observed in our study. Recent results characterizing tick-pathogen molecular interactions support the hypothesis that *A. phagocytophilum* has evolved common mechanisms to establish infections in tick vectors and vertebrate hosts, due to its adaptation to a variety of vector and reservoir species [7]. The mechanisms that *A. phagocytophilum* uses to infect and replicate in tick and vertebrate hosts are still largely unknown, and the interactions between different genetic variants of *A. phagocytophilum* are not fully explored [35]. In our study, we found ticks infected with *A. phagocytophilum* (*groEL* ecotype II/*ankA* clusters 2, 4 and 7) that are not known to be responsible for HGA in Europe. However, Kim et al. [36] reported a single clinical case, caused by *A. phagocytophilum* (in our study: ecotype II/cluster IV) from South Korea, where the pathogen is transmitted by ticks other than *I. ricinus*. Strains with zoonotic potential known from Europe represent only a small fraction of the *A. phagocytophilum* genetic variants and belong to *ankA* cluster 1/*groEL* ecotype I. These monophyletic groups are characterized by a wide host range, including domestic and wild animals, often with a synanthropic lifestyle or living closely to human settlements (e.g. foxes, hedgehogs, wild boars). The lack of reported clinical cases caused by *A. phagocytophilum* strains other than ecotype I/cluster 1 in Europe may therefore be related to their low prevalence in the environment, and their ability to infect humans needs to be confirmed.

Notwithstanding the noted variability of *A. phagocytophilum*, this tick-borne pathogen is considered to be a single species. This contrasts with a similar situation in *B. burgdorferi* sensu lato, which is subdivided into several operational taxonomic units (referred to as genospecies) with differences in geographic distribution, host species preferences, zoonotic potential and tissue tropism [37]. The introduction of a comprehensive and unified nomenclature and tools defining genetic lineages of *A. phagocytophilum* would allow easier communication of results and deeper understanding of the ecology and epidemiology of different genetic variants of this bacterium.

## Supplementary Information

**Additional file 1: Table S1.** Primers used in the study.

**Additional file 2: Figure S1.** Detailed maximum likelihood phylogenetic tree based on the groEL gene (*groEL*) of *A. phagocytophilum.*

**Additional file 3: Figure S2.** Detailed Maximum likelihood phylogenetic tree based on the ankA gene (*ankA*) of *A. phagocytophilum*.

## Data Availability

Representative sequences of *groEL* and *ankA* were deposited to the NCBI GenBank database under accession numbers OP265397-OP265406 and OP265407-OP265426, respectively. They will not be released to the public database until 18 Sep 2023, or until the data or accession numbers appear in print, whichever is first.
