# Supplementary material for: Distant genetic variants of Anaplasma phagocytophilum from Ixodes ricinus attached to people
Source: Parasit Vectors. 2023 Feb 28;16:80. doi: 10.1186/s13071-023-05654-y (PMC9976488; doi:10.1186/s13071-023-05654-y)
Supplement: Supplementary file 1 — Additional file 1: Table S1. Primers used in the study. [file 13071_2023_5654_MOESM1_ESM.docx]

Table S1. Primers used in the study

| **Target gene** | **Primer name** | **Primer sequence (5’ - 3’)** | **Annealing temperature /product length (bp)** | **Reference** |
| --- | --- | --- | --- | --- |
| *groESL* | EphplgroEL(569)F | ATGGTATGCAGTTTGATCGC | 57°C/574 | (Alberti et al. 2005) |
|  | EphgroEL(1142)R | TTGAGTACAGCAACACCACCGGAA |  |  |
|  | ApNest-F | GTGGAATTTGAAAATCCATAC | 55°C/407 | (Jaarsma et al. 2019) |
|  | ApNest-R | GTCCTGCTAGCTATGCTTTC |  |  |
|  | HS1-modif | TGGGCTGGTARTGAAWT | 48°C/1380 | (Liz et al. 2002) |
|  | HS6 | CCICCIGGIACIAYACCTTC |  |  |
|  | HS43-modif | ATAGCTAAGGAAGCATAGTC | 52°C/1297 |  |
|  | HSVR | CTCAACAGCAGCTCTAGTAGC |  |  |
| *ankA*  *2* | ankA_eF | TGAGCCTCACCCGCAGCATG | 63°C /500 | (Chastagner et al. 2013) |
|  | ankA_eR | CTCTGCGTTGCTGGAGCCCC |  |  |
|  | ankA_iF | CTCACCCGCAGCATGTTG | 57°C/490 |  |
|  | ankA_iR | GTTGCTGGAGCCCCTTTATCC |  |  |
| *ankA*  *1* | SLO fo 1 | GGGATRAGTGCRGTGCAGYAT | 52°C/1109 | (Huhn et al. 2014) |
|  | SLO re 1 | TACTGCRGCMGCTARAGGRCT |  |  |
|  | SLO fo 2 | ACTGCRGCMGCTARAGGRCT | 52°C/523 |  |
|  | SLO re 2 | AWRGWTCCSKYAGGAGYATTTA |  |  |
