# Supplementary figures and images for: Distant genetic variants of Anaplasma phagocytophilum from Ixodes ricinus attached to people
Source: Parasit Vectors. 2023 Feb 28;16:80. doi: 10.1186/s13071-023-05654-y (PMC9976488; doi:10.1186/s13071-023-05654-y)

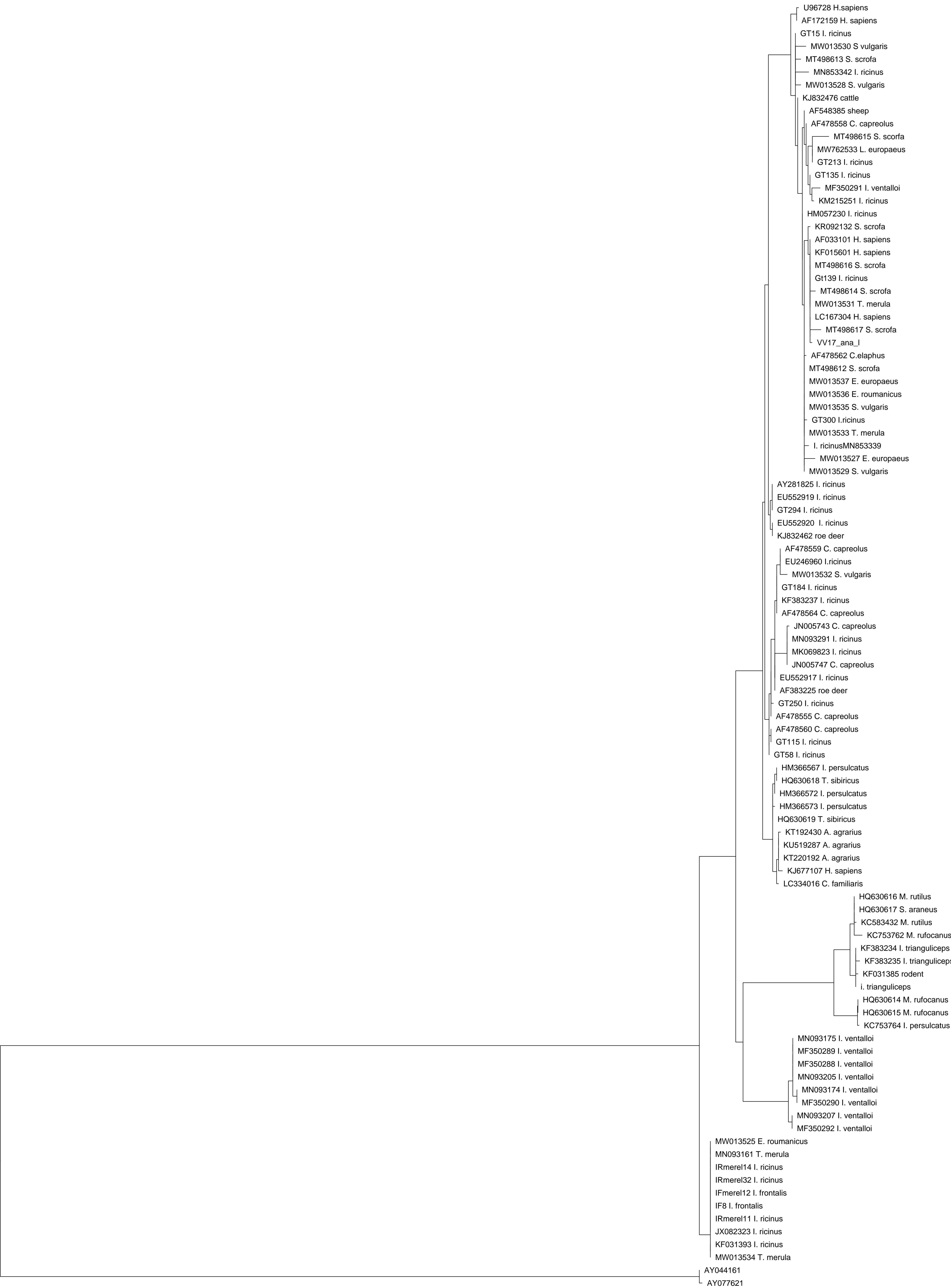

Supplement: Supplementary file 2 — Additional file 2: Figure S1. Detailed maximum likelihood phylogenetic tree based on the groEL gene (groEL) of A. phagocytophilum. [file 13071_2023_5654_MOESM2_ESM.pdf]

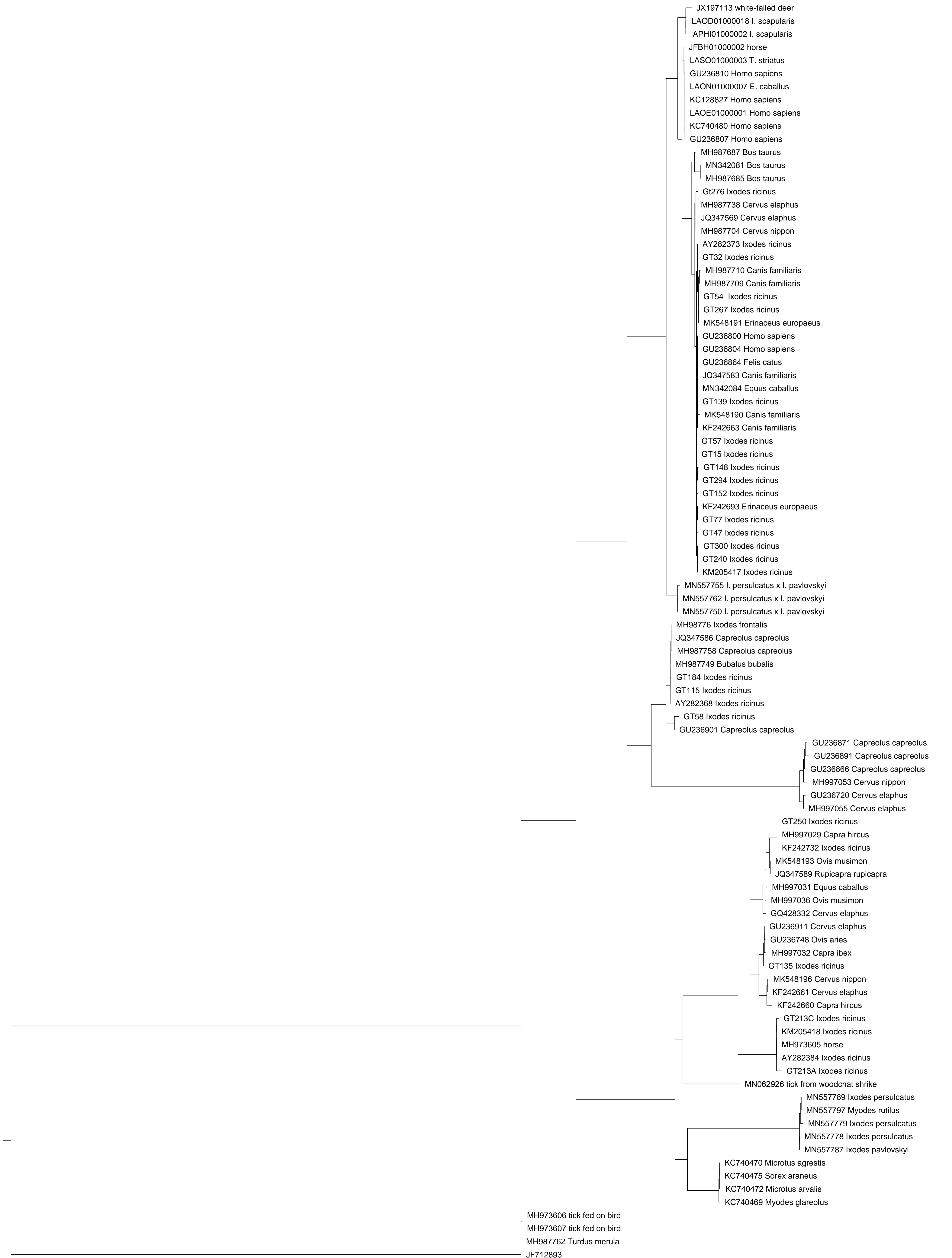

Supplement: Supplementary file 3 — Additional file 3: Figure S2. Detailed Maximum likelihood phylogenetic tree based on the ankA gene (ankA) of A. phagocytophilum. [file 13071_2023_5654_MOESM3_ESM.pdf]
